# Is Inferior Alveolar Nerve Block Sufficient for Routine Dental Treatment in 4- to 6-year-old Children?

**DOI:** 10.5005/jp-journals-10005-1467

**Published:** 2017-02-27

**Authors:** Maryam Pourkazemi, Leila Erfanparast, Sanaz Sheykhgermchi, Milad Ghanizadeh

**Affiliations:** 1 Assistant Professor, Department of Pediatric Dentistry, Tabriz University of Medical Sciences, Tabriz, Islamic Republic of Iran; 2 Assistant Professor, Department of Pediatric Dentistry, Tabriz University of Medical Sciences, Tabriz, Islamic Republic of Iran; 3 Consultant, Department of Pediatric Dentistry, Tabriz University of Medical Sciences, Tabriz, Islamic Republic of Iran; 4 Postgraduate Student, Department of Oral and Maxillofacial Surgery, Faculty of Dentistry, Tabriz University of Medical Sciences, Tabriz, Islamic Republic of Iran

**Keywords:** Anesthetized extent, Buccal gingiva, Local anesthesia, Long buccal nerve, Primary dentition.

## Abstract

**Introduction:**

Pain control is one of the most important aspects of behavior management in children. The most common way to achieve pain control is by using local anesthetics (LA). Many studies describe that the buccal nerve innervates the buccal gingiva and mucosa of the mandible for a variable extent from the vicinity of the lower third molar to the lower canine. Regarding the importance of appropriate and complete LA in child-behavior control, in this study, we examined the frequency of buccal gingiva anesthesia of primary mandibular molars and canine after inferior alveolar nerve block injection in 4- to 6-year-old children.

**Study design:**

In this descriptive cross-sectional study, 220 4- to 6-year-old children were randomly selected and entered into the study. Inferior alveolar nerve block was injected with the same method and standards for all children, and after ensuring the success of block injection, anesthesia of buccal mucosa of primary molars and canine was examined by stick test and reaction of child using sound, eye, motor (SEM) scale. The data from the study were analyzed using descriptive statistics and statistical software Statistical Package for the Social Sciences (SPSS) version 21.

**Results:**

The area that was the highest nonanesthetized was recorded as in the distobuccal of the second primary molars. The area of the lowest nonanesthesia was also reported in the gingiva of primary canine tooth.

**Conclusion:**

According to this study, in 15 to 30% of cases, after inferior alveolar nerve block injection, the primary mandibular molars’ buccal mucosa is not anesthetized.

**How to cite this article:** Pourkazemi M, Erfanparast L, Sheykhgermchi S, Ghanizadeh M. Is Inferior Alveolar Nerve Block Sufficient for Routine Dental Treatment in 4- to 6-year-old Children? Int J Clin Pediatr Dent 2017;10(4):369-372.

## INTRODUCTION

Pain control is one of the most important aspects of behavioral control in children,^1^ and the most common method used to achieve pain control in dental procedures is using LA.^2^ Local anesthetic is obtained by a correct and accurate injection that not only comforts the patient and reduces pain, but also increases patients’ trust in the dentist.^3,4^ The most common method of anesthesia in the mandible is inferior alveolar nerve block.^5^ Incisive, mental, and lingual nerve (in most cases) are branches of the inferior alveolar nerve anesthetized following inferior alveolar nerve block injection, but the long buccal branch is not anesthetized in this method.^6,7^ So, in cases where surgery is done on the soft tissue on the buccal surface of the mandibular permanent molars, immediately after inferior alveolar nerve block injection, buccal nerve should be anesthetized.^7-9^ The buccal nerve (N. buccalis) is the sensory branch of anterior division of mandibular nerve, passing between lateral pterygoid muscles. It crosses the anterior border of the ramus of mandible at a similar level to the lower third molar and distributes to cheek’s soft tissue.^9^ Buccal nerve block is useful for reducing buccal soft tissue pain for various dental procedures, such as placing a dam clamp, removal of subgingival caries, sub-gingival tooth preparation, gingival cord retraction, and operating on abnormal lesions or infection of mandible buccal mucosa.^9,10^ Many studies revealed that the buccal nerve innervates buccal gingiva and mandible mucosa for a variable range from the vicinity of the lower third molar to the lower canine.^9-13^ According to the report of Bahl,^2^ long buccal nerve innervates buccal mucosa and gingiva adjacent to teeth of mandibular molars and second premolars. Other study that was carried out by Wongsirichat et al^9^ on the 20- to 60-year-old people indicated that distobuccal and midbuccal of second premolar is innervated by long buccal nerve in 27.5 and 12.5% of cases respectively. They also indicated that distobuccal and midbuccal of primary premolar is innervated by long buccal nerve in less frequency. Many studies show that long buccal nerve innervates the mandibular buccal mucosa and gingival with a variable pattern, and due to the importance of appropriate and complete local anesthesia in children’s behavior management and lack of similar study, we examined the frequency of buccal gingiva anesthesia of primary mandibular molars and canine following inferior alveolar nerve block injection in 4 to 6-year-old children. The results of this study can be attributed to achieving desirable local anesthesia and reduction of children’s behavior problem.

## MATERIALS AND METHODS

The participants included 220 healthy children (107 boys and 113 girls) aged 4 to 6 years, enrolled in the Department of Pediatric Dentistry, Tabriz University of Medical Sciences, Tabriz, Islamic Republic of Iran, during the period from January to June 2015. The participants were mostly referrals from the general practitioners to the Department of Pediatric Dentistry. The selected subjects were in complete physical and mental health, with no confounding medical history.

The following criteria were considered for inclusion in the study: Need for inferior alveolar nerve block for routine dental treatment; no contraindication for administration of local anesthesia (lidocaine with epinephrine); no inflammatory lesions in primary molars buccal mucosa; children with previous inferior alveolar nerve block experience; and children who were in the rate of 3 or 4 of Frankel behavior rating scale. Study populations were randomly selected and they participated in the study. The study procedure was explained to the parents and an informed written consent was taken. The study procedure was approved by the Research and Ethics Committees of the Tabriz University of Medical Sciences.

The inferior alveolar nerve blocks were injected by the pediatric dentist. The patients were injected with 2% (lidocaine hydrochloride with epinephrine 1/80,000) in the amount of 1 mL with accepted technique for children. The needle (27 gauge and 20 mm) was inserted at the level of occlusal plane anterior to pterygomandibular raphe at a depth of approximately 5 mm.

The barrel of the syringe was directed on a plane between the two primary molars on the opposite side of the arch (McDonald). About 5 minutes after the patients were injected, ensuring the success of block injection by controlling anesthesia around the lower lip, anesthesia was detected at each point at primary canine and molars buccal gingival with a sharp probe calibrated by instrument weight about 20 to 40 gm at the same side.^5-7,14^ For this purpose, the buccal gingival of primary molars was examined in three mesiobuccal (3 mm below the free gingival margin along with mesiobuccal line angle), midbuccal (3 mm below the free gingival margin along the buccal groove), and distobuccal (3 mm below the free gingival margin along with distobuccal line angle) of primary molars. Buccal gingiva of primary canine was examined in midbuccal respectively. Totally, 37 out of 220 children participating in the study (16.8%) had the first permanent molar. The child’s reaction to pain was assessed by SEM scale designed by Wright et al in which the subject’s response is graded on a scale from 1 to 4. Reaction in the rates of 2, 3, and 4 on this scale were recorded as nonanesthetized.^15^ Data collection instrument was a checklist. All cases were examined by one examiner. The k value of intraexaminer agreement was 0.97. Data were analyzed using descriptive statistics and chi-squared test and software SPSS version 21.

## RESULTS

**Graph 1:**
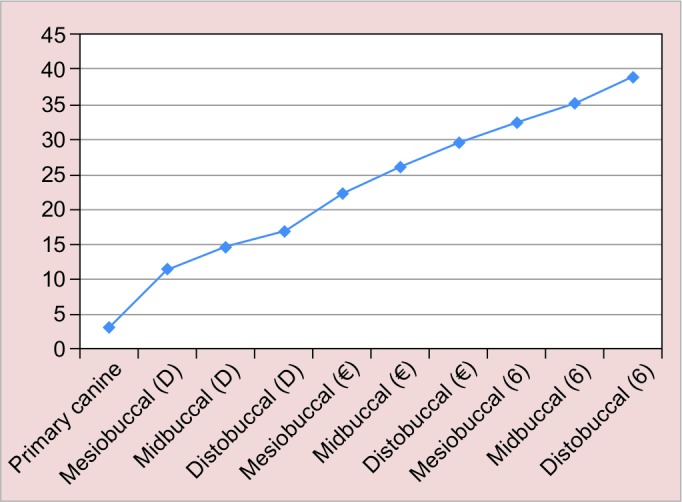
Frequency of nonanesthetized areas of buccal gingiva of primary molars, canines, and first permanent molars

The percentage of anesthetized and nonanesthetized areas after inferior alveolar nerve block in 4 to 6-year-old children is shown in Table 1 and Graph 1.

**Table 1:** **Table 1:** Frequency of anesthetized and nonanesthetized areas of buccal gingiva of primary canines and molars

						*Nonanesthesia*		*Anesthesia*	
Primary canine				No.		8		212	
				%		3.2		96.8	
First primary molars		Mesiobuccal		No.		25		195	
				%		11.4		88.6	
		Midbuccal		No.		32		188	
				%		14.5		85.5	
		Distobuccal		No.		37		183	
				%		16.8		83.2	
Second primary molars		Mesiobuccal		No.		49		171	
				%		22.3		77.7	
		Midbuccal		No.		57		163	
				%		25.9		74.1	
		Distobuccal		No.		65		155	
				%		29.5		70.5	

**Table 2:** **Table 2:** Frequency of anesthetized and nonanesthetized areas of buccal gingiva of first permanent molars

*First permanent molars*				*Nonanesthesia*		*Anesthesia*	
Mesiobuccal		No.		12		25	
		%		32.4		67.6	
Midbuccal		No.		13		24	
		%		35.1		64.9	
Distobuccal		No.		14		23	
		%		38.9		61.1	

The frequency of nonanesthetized areas of buccal gingiva from canine to the distobuccal of second primary molar was increased. The highest percentage of nonanes-thetized areas in primary dentition were in the disto-buccal gingiva of second primary molar (29.5%). While the percentage of nonanesthetized area was the least in canine gingiva (3.2%). As an adjunctive finding, it was seen that after inferior alveolar nerve block in children who had first permanent molar (37 out of 220), some areas of gingiva were not anesthetized (Table 2 and Graph 1).

No difference in the distribution of area of anesthesia between sexes was found (p-value > 0.05).

## DISCUSSION

Regarding local analgesia in dentistry, many studies on long buccal nerve block have revealed that buccal nerve has a variable pattern of innervation from buccal gingiva and mandible mucosa of the lower third molar to the canine.^9-13^ No study has been done to examine the frequency of mandibular gingiva anesthesia after inferior alveolar nerve block injection in children. Thus, in this study, we examined the frequency of buccal gingival anesthesia of canines and primary mandibular first and second molars after inferior alveolar nerve block injection without long buccal nerve block in children 4 to 6 years old. According to our observations, despite the success of inferior alveolar nerve block, buccal gingival was not anesthetized in 1/6 of first and 1/3 of second primary molars, and frequency of nonanesthetized area was increased from canine to the first permanent molar gingiva. It could be concluded that these areas are innervated by the long buccal nerve. Wongsirichat et al^9^ in a study examined the extent of long buccal nerve in adults with a mean age of 30 years and concluded that buccal nerve intervened gingiva and mandibular mucosa with a varied pattern. This is more than 80% between the retromolar area to the second molar. The first molar to the second premolar had more decrement (80-20%), but least (less than 20%) from the first premolar to the central incisor. Blanton and Jeske^11^ study also showed that the long buccal nerve innervates mandibular buccal gingival in a varied pattern from the third molars to canines. Carter and Keen^16^ and Coleman and Smith^17^ in a separate study reported that buccal nerve innervates buccal gingival mucosa of molars and retromolar site in adults. According to the report of Bahl,^2^ long buccal nerve innervates buccal mucosa and gingiva adjacent to mandible permanent molar and second premolars. However, regarding the results of the different studies, it seems that buccal gingiva innervation varied in different populations. Given the importance of adequate anesthesia in dental operations and the importance of pain control in the behavior management in pediatric dentistry, for mandibular primary molars and canine region, after the inferior alveolar nerve has been blocked, the dentist should check for complete anesthesia of the soft tissue before the operations, such as placement of clamps, matrix band, wedge, and extraction, and might consider supplemental injection of the buccal nerve for more complete anesthesia of the buccal gingiva.

## CONCLUSION

According to the study, 15 to 30% of cases after inferior alveolar nerve block injection of primary mandibular molars buccal mucosa are not anesthetized. We hope dentists by knowledge of this point would control buccal anesthesia and, if required, consider supplementary injections.
